# Multicenter randomized controlled trial of exercise in aortic dissection survivors: rationale, design, and initial hemodynamic data

**DOI:** 10.20517/2574-1209.2023.149

**Published:** 2024-05-15

**Authors:** Yasmin A. Toy, Kayla N. House, Leslie M. Boyer, Jennifer L. McNamara, Marion A. Hofmann-Bowman, Kim A. Eagle, Michelle S. Lim, Alan C. Braverman, Siddharth K. Prakash

**Affiliations:** 1 Department of Internal Medicine, John P. and Kathrine G. McGovern Medical School, University of Texas Health Science Center at Houston, Houston, TX 77030, USA.; 2 John T. Milliken Department of Medicine, Washington University School of Medicine in St. Louis, St. Louis, MO 63110, USA.; 3 Department of Internal Medicine, University of Michigan Medical School, Ann Arbor, MI 48109, USA.

**Keywords:** Exercise, thoracic aortic dissection, thoracic aortic aneurysm, heritable thoracic aortic disease, ambulatory blood pressure monitoring, hypertension

## Abstract

**Aims:**

There are currently no evidence-based guidelines for exercise after thoracic aortic dissection (TAD), leading to highly variable recommendations that frequently lead patients to restrict their physical activities. This multicenter randomized controlled trial was intended to evaluate the safety and efficacy of a moderate intensity guided exercise program for TAD survivors.

**Methods:**

Participants were eligible if they had a Type A or Type B dissection at least 90 days before enrollment and could attend two in-person study visits. The guided exercise circuit consisted of six aerobic, isotonic, or isometric exercises that participants continued at home with virtual follow-up sessions. The primary endpoint is the change in the composite anxiety and depression PROMIS-29 T-score at 12 months. Secondary endpoints include changes in grip strength, weight, 24-h ambulatory blood pressure, and arterial biomechanical properties measured by central arterial waveform analysis.

**Results:**

Preliminary analysis of the first 81 enrolled participants demonstrated that the guided exercise circuit was completed safely and was not associated with severe hypertension, injury, or adverse cardiovascular events. At enrollment, adverse central waveform or ABPM characteristics were prevalent and were significantly associated with exertional hypertension.

**Conclusions:**

Guided exercise is safe for aortic dissection survivors. Follow-up of enrolled participants will conclude in October 2024.

## INTRODUCTION

Thoracic aortic dissection (TAD) is a life-threatening medical emergency caused by an intimal tear in the thoracic aortic wall^[1]^. Although advances in the surgical intervention, prophylaxis, and early recognition of TAD have improved long-term survival, many individuals with TAD face disabling, lifelong obstacles related to anxiety about sudden risk of death, re-dissection, aortic rupture, and other chronic complications^[2,3]^.

Observational studies have documented decreased quality of life in TAD survivors related to changes in daily activity levels and frequent anxiety or depression^[3,4]^. Case reports about acute aortic dissections that occurred during high-intensity exercises such as weightlifting have provoked uncertainty about the safety of exercises^[5–7]^. Weightlifting may induce extreme elevations in blood pressure due to potent pressor and Valsalva responses^[8]^. Anxiety about dissection risk may lead TAD survivors to minimize physical activities, leading to additional deterioration of overall cardiovascular health and mental well-being^[3]^. There is an urgent need to clarify the safety and benefits of exercise^[9]^. TAD survivors may also benefit from accessible, personalized interventions that address mental health issues^[1]^.

Physical activities can provide synergistic benefits in combination with antihypertensive medications, reducing morbidity and mortality across a wide spectrum of cardiovascular diseases. Regular aerobic or isometric exercise is associated with a dose-dependent decrease in blood pressure, major cardiovascular outcomes, and mortality^[10]^. On a weekly basis, at least 150 min of moderate aerobic exercise and 20 min of isometric exercise can reduce systolic blood pressure^[11]^. Moderate aerobic activity decreased aortic medial degeneration and reduced aortic dilation in a Marfan syndrome mouse model^[6]^. Abdominal aortic aneurysm expansion rates decreased in patients who engaged in moderate intensity exercise, especially when coupled with improved control of systolic hypertension^[12]^. Cardiac rehabilitation has been proved to be safe and effective in post-surgical TAD patients, resulting in increased aerobic capacity and quality of life^[12–14]^. However, only one-quarter of eligible patients in the United States have access to cardiac rehabilitation programs due to social and economic barriers, depriving many patients of the proven benefits related to early guided exercise after surgery or dissection^[15]^. Therefore, exercises that can be performed at home with inexpensive and portable equipment are needed to improve access to the benefits of cardiac rehabilitation.

Regular exercise may uniquely benefit TAD survivors by improving mental health, quality of life, and functional capacity^[3]^. The overall goal of this study is to determine if a guided exercise program consisting of static and dynamic maneuvers that can be performed at home can decrease anxiety and increase confidence to engage in physical activities while lowering systolic blood pressure, arterial stiffness, and other cardiometabolic health measures^[11]^. We demonstrate how this reproducible protocol can be used to improve the mental and physical well-being of TAD patients.

## METHODS

### Ethics

The study protocol was reviewed and approved by the Committee for the Protection of Human Subjects at the University of Texas Health Science Center at Houston (UTHealth Houston), University of Michigan, and Washington University School of Medicine in St. Louis. The protocol number is HSC-MS-22–0936 and the approval date was November 28, 2022. All subjects signed a written informed consent document prior to enrollment.

### Inclusion/exclusion criteria

**Figure 1.**
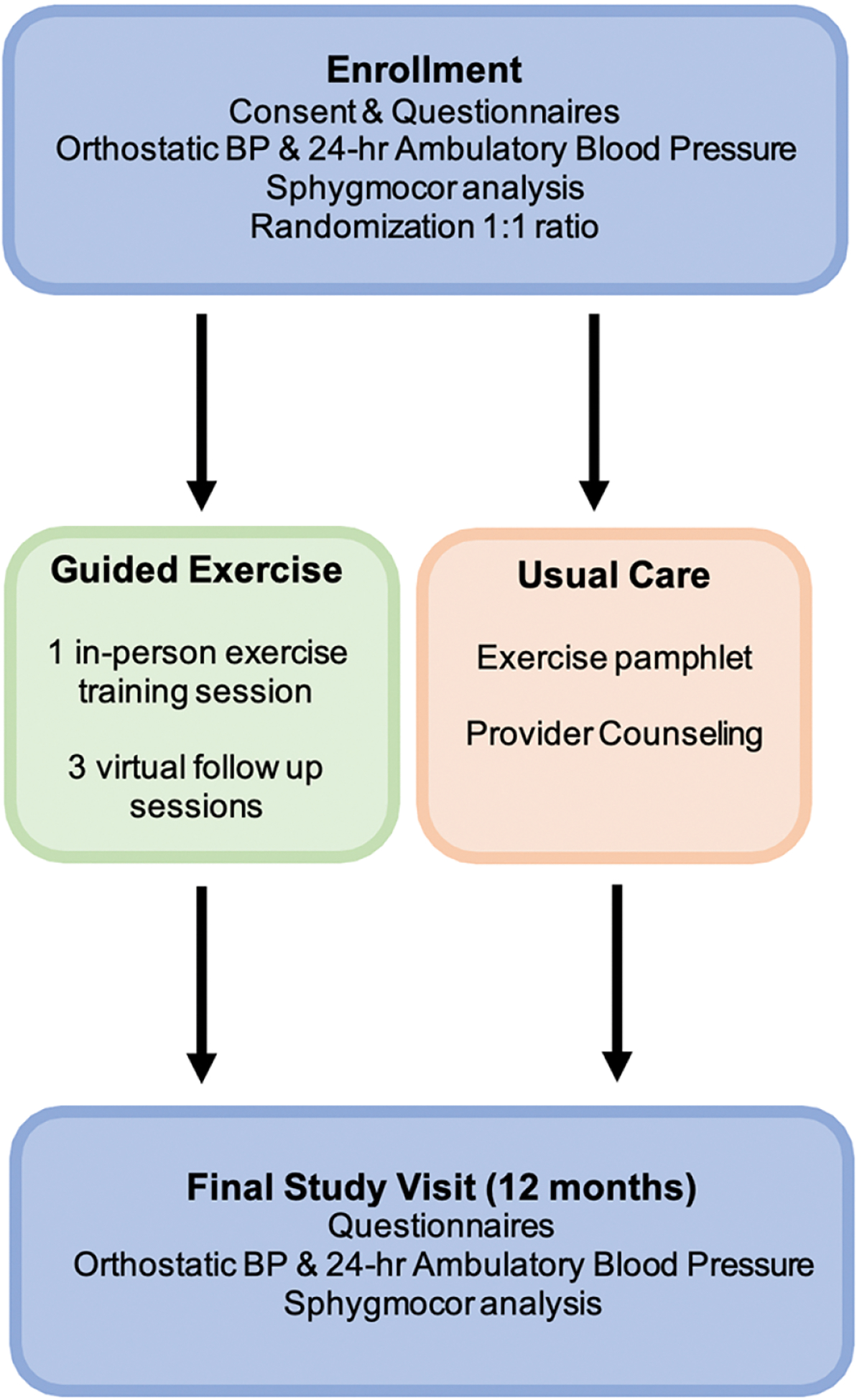
Clinical trial design and workflow for guided exercise and usual care groups. Guided exercise participants returned surveys about their routine activities and/or exercise diaries at each study visit.

This nonblinded randomized controlled clinical trial (clinicaltrials.gov NCT05610462) was designed to proceed for 18 months: an anticipated 6 months to complete enrollment (May-September 2023) and 12 months of follow up for each participant [Figure 1]. Aortopathy clinics at UTHealth Houston, Washington University in St. Louis, and the University of Michigan will recruit a total of 126 patients (42 at each site), male and female, through clinician referral, medical records, databases, and social media campaigns. Patients who survived a thoracic aortic dissection (Type A or B) at least three months prior to study enrollment were eligible for inclusion. All potential participants were required to complete the 2009 Behavioral Risk Factor Surveillance Survey (BRFSS) about the amount of time spent weekly on moderate and strenuous physical activities [Supplementary Text 1]. Patients were excluded if any of the following apply: routine participation in greater than 150 min per week of moderate-intensity exercises (as assessed by the BRFSS); unable to attend at least one exercise training session in person; uncontrolled hypertension (mean SBP greater than 160 mmHg at rest); symptomatic aortic, coronary, or vascular disease; unable to complete exercise program due to physical limitations, equipment or space limitations, or time commitment; do not own a treadmill or stationary cycle or have regular access to one at a gym. If patients are participating in cardiac rehabilitation, enrollment is delayed until after discharge from the rehabilitation program.

### Study design

After confirmation of eligibility and consent, all participants completed a demographic survey (age, sex, race, ethnicity), and the PROMIS-29 v2.0 profile questionnaire (Patient-Reported Outcomes Measurement Information System), which is validated to assess seven health domains (physical function, fatigue, pain, depressive symptoms, anxiety, ability to participate in social roles and activities, and sleep disturbance) [Supplementary Text 2]. At enrollment, all participants were fitted with ambulatory blood pressure monitors to wear for 24 h (ABPM, OnTrak, Space Labs, Inc.) with the cuff on the non-dominant arm. We performed arterial pressure waveform and pulse wave velocity analysis (Sphygmocor, AtCor Medical, Inc.) and recorded one set of orthostatic vital signs (sitting × 3, supine, standing). We also obtained consent to extract additional outcome data from health records. The study team at UTHealth Houston (KH) randomly allocated participants in a 1:1 ratio to receive the guided exercise program or usual care on the day of enrollment. Randomization was stratified according to sex and enrollment site using the randomization module in REDCap. Participants in both study arms received all usual clinically indicated care, including diagnostic tests and medications. Recommendations for tests or interventions did not change based on the assigned study arm. At the concluding study visit 12 months after enrollment, all study participants will repeat blood pressure measurements and complete the same BRFSS activity and PROMIS-29 v2.0 questionnaires as at enrollment.

### Guided exercise

At enrollment, participants who were randomized to the guided exercise arm completed a supervised exercise protocol that included two circuits of six moderate-intensity exercises: bicep curls, wall sits, hand grips, leg raises, stationary cycling and treadmill. Bicep curls were performed with the dominant hand using 5-, 8-, or 10-pound weights. Wall sits were maintained with a ninety-degree angle between the back and lower legs. Hand grip resistance level was calculated as 40% of maximal exertion using the dominant hand. Leg raises were performed in a supine position, with both heels elevated six inches above ground level. Stationary cycling was performed at a target of 100 Watts. The treadmill was performed once at 3 mph at a 14% grade incline. Cardiac rehabilitation facility staff and trained study personnel supervised all exercises. Participants maintained exercises at moderate intensity (50%–80% of age-adjusted maximum heart rate) long enough to acquire one brachial cuff reading (1–2 min). Blood pressure measurements during exercise were manually triggered using the ABPMs and supervisors ensured that the measurement arm was immobilized while the cuff inflated as recommended in the AHA scientific statement on blood pressure measurement^[16]^. All exercises were initiated for 15 s prior to triggering the ABPM and maintained until the readings were completed. Post-exercise blood pressure measurements were taken following each exercise. Exertional hypertension was defined as any systolic blood pressure > 180 mmHg or diastolic blood pressure > 100 mmHg on more than 1 exercise. Exercises were promptly terminated if any of the following occurred: persistent systolic pressure > 160 mmHg persisting after 3 min of recovery, any single systolic pressure > 210 mmHg, or any single diastolic blood pressure > 120 mmHg; chest pain, dyspnea, or significant fatigue; or a request to stop. Perceived exertion during each exercise was measured using the Borg CR-10 scale, with a score of one representing minimal exertion and a score of ten indicating maximal exertion^[17]^. Participants received individualized instruction about how to implement the exercise program at home, with the weekly target of 5 days or at least 150 total min of moderate-intensity exercise. They were also counseled to record their activities in a monthly exercise diary and share fitness data recorded by home blood pressure cuffs or wearable devices.

After enrollment, the study teams followed up on participants via video check-ins and surveys. All participants completed BFRSS surveys about the intensity and frequency of their activities at 1, 3, and 9 months, and at the conclusion of the study. In the first month after enrollment, the study teams conducted one video check-in with each participant to assess any changes in health status, obtain information about clinic visits, track exercise progress, reinforce teaching about the exercise circuit, and answer questions about exercise. During the check-in, the study team also observed and corrected participants as they performed one exercise. Shorter video visits without exercise demonstrations were repeated at 3 and 9 months after enrollment, in which participants were encouraged to share their experiences with the exercise protocol and to troubleshoot potential obstacles to exercise. Participants also transmitted home blood pressure or fitness data to UTHealth if they were available. The study teams promoted the target of more than 150 min of moderate exercises per week at each interaction.

### Usual care

Participants who were randomized to usual care completed 24-h ABPM but did not receive any teaching regarding exercise and did not participate in any in-person or virtual exercise sessions. Instead, they attended routine clinic visits and received standardized counseling about exercise, including a pamphlet with guidelines about living with aortic disease. Participants were not contacted by the study team after the initial enrollment visit.

### Data analysis

The primary outcome is a clinically significant change in the PROMIS-29 T score or the PROMIS mental health summary score, a subset of PROMIS questions that primarily assess emotional distress (anxiety and depressive symptoms). The general population mean of PROMIS T scores is standardized at 50 points with a standard deviation of 10 points. The minimum clinically important difference (CID) is 5 points. To detect a change in 5 T score points with beta = 0.80 and alpha = 0.05, target sample size is 63 patients per study arm (126 total).

ABPM outcomes included mean 24-h, daytime, and nocturnal blood pressures. Pulse pressure, nocturnal dipping status, blood pressure variability, AASI, and peak systolic pressure were also included in the analysis because they have been identified as independent predictors of cardiovascular mortality^[18,19]^. Study thresholds were derived from published data on ABPM norms: mean 24-h pressure > 125/75 mmHg, mean daytime pressure > 130/80 mmHg, mean nighttime pressure > 110/65 mmHg, ambulatory arterial stiffness index (AASI) ≥ 0.70, nocturnal dipping < 10%, peak daytime systolic pressure >180 mmHg, 24-h pulse pressure > 53 mmHg, and increased blood pressure variability, defined as a coefficient of variation > 11.1. Postural orthostasis was defined as a > 20 mm Hg decrease in systolic blood pressure and/or a > 10 mm Hg decrease in diastolic blood pressure when sitting or standing from a supine position. ABP data were analyzed using Sentinel software (v11, Space Labs, Inc., Snoqualmie, WA). Multiple comparisons were assessed using one-way ANOVA with the Tukey method.

## RESULTS

### Study cohort

A total of 445 individuals were screened and 250 were found to be eligible for the study. The major reasons why individuals were excluded were: unable to attend in-person study visits (55), exercise equipment inaccessibility (49), and physical inability to exercise (42). To date, a total of 81 trial participants were enrolled with complete study data [Table 1]. No participants were excluded after randomization. Participants who had Type A dissections (*n* = 51) received TEVAR (*n* = 2), had open repairs (*n* = 44), or had no interventions (*n* = 5). Participants who had Type B dissections (*n* = 25) received TEVAR (*n* = 10), had open repairs (*n* = 4), or had no interventions (*n* = 11). Participants who had more than one dissection underwent only open repairs (*n* = 5). There were no significant differences in demographic characteristics by intervention type [Supplementary Table 2].

### PROMIS questionnaire

Evaluation of seven PROMIS domains found that mean T scores for anxiety (51 ± 9), pain (51 ± 7), and impairment of participation in social activities (54 ± 8) were increased. Scores for depression, fatigue, and sleep disturbance were within normal limits. There were no significant differences between PROMIS scores for participants with and without exertional hypertension, between guided exercise and control groups, or by intervention type [Supplementary Material].

### Grip strength

At baseline, the mean maximum grip strength was 64 lbs (IQR 12.9). At the first follow-up visit, grip strength increased by a mean of 7.8 lb (IQR 6.9). There were no significant differences in grip strength by intervention type [Supplementary Material].

### Orthostatic and ambulatory blood pressure

At baseline, seven participants (9%) exhibited postural orthostasis. The most prevalent adverse ABPM characteristics were nocturnal hypertension (83%), blunted nocturnal dipping (40%), and elevated mean 24-h pulse pressure (40%). Participants who developed significant exertional hypertension had higher peak blood pressure values and greater ambulatory blood pressure variability [Table 2]. There were no significant differences in ABPM characteristics when participants were stratified by intervention type [Supplementary Table 3]. There was no association between postural orthostasis and exertional hypertension.

### Safety of exercise protocol

All participants completed the study protocol. To date, there have been no participant deaths during the trial observation period. One in-person exercise session was temporarily delayed after a participant developed severe exertional hypertension (SBP > 210), but they were able to complete the protocol after medication adjustment. Exercises that caused SBP to exceed 180 mmHg were: bicep curls (3/37, 8%), wall sits (7/35, 20%), hand grips (1/37, 3%), leg raise (1/37, 3%), stationary bicycling (4/32, 13%), and treadmill (3/36, 8%). Exercises that caused DBP to exceed 100 mmHg were: bicep curls (5/37, 14%), wall sits (11/35, 31%), hand grips (7/37, 19%), leg raise (6/37, 16%), stationary bicycling (1/32, 3%), and treadmill (3/36, 8%). There were no significant differences in the prevalence of exertional hypertension when participants were stratified according to intervention type [Supplementary Material].

### Participant feedback

A total of 35 participants returned surveys about their enrollment experiences (see Supplementary Text 3 for survey details). More than half of respondents (*n* = 23) agreed that participating in the clinical trial improved their outlook on exercise. Participants expressed increased confidence to engage in physical activities and optimism about participating in exercise after attending an in-person clinical trial visit.

### Technical issues

Mean individual exercise completion rates from highest to lowest were bicep curls (100%), hand grip (100%), leg raise (100%), treadmill (97%), wall sit (95%), and cycle (87%). The rates for exercises with two readings per participant from highest to lowest were hand grip (92%), leg raise (92%), treadmill (87%), wall sit (84%), bicep curls (82%), and cycle (76%). The most frequent ABPM errors corresponded to excessive arm movement or vibration [Supplementary Table 1]. Bracing the measurement arm successfully suppressed most of these errors^[16]^. We addressed the other major sources of error by changing ABPM cuff size or refitting cuffs according to the manufacturer’s guidelines. To limit overexertion, we attempted ABPM blood pressure readings a maximum number of two times before switching to manual auscultation.

## DISCUSSION

Anxiety and uncertainty about exercise may negatively impact the cardiovascular and mental health of TAD survivors by leading them to restrict their activities. In contrast to case reports that inform current guideline recommendations, this pilot study is the first randomized controlled trial of exercise in TAD survivors. The unique objectives of this study are to assess the effects of an at-home exercise program on hemodynamic and mental health outcomes. The primary outcome is a clinically significant change in the PROMIS-29 summary T-score or mental health summary score. Secondary outcomes include the change in the burden of ambulatory hypertension and nocturnal dipping as assessed by ambulatory blood pressure monitoring. The guided exercise program was proved to be safe for trial participants, and we found that grip strength, a significant predictor of cardiovascular death, increased by 30% in the first three months of participation^[20,21]^. We also observed adverse ABPM characteristics in many participants that are associated with increased cardiovascular mortality, such as nocturnal hypertension, blunted nocturnal dipping, or elevated pulse pressure. Ambulatory peak blood pressure and blood pressure variability predicted significant exertional hypertension. These observations highlight the high cardiovascular risk of the trial cohort. While self-reported anxiety was increased in trial participants, there was no correlation between initial PROMIS anxiety T-scores and ambulatory or exertional hypertension.

As the study progressed, we made several adjustments to home exercise instructions and the virtual visit protocol to account for the frailty and decreased physical strength of many TAD participants. The exercise instructions were altered so that participants were able to maintain moderate intensity effort without physical strain. Participants were instructed to scale up individual exercises incrementally, by increasing repetitions in 15-s increments or by two repetitions per week. When starting home exercises, we allowed participants to decrease the initial speed and incline settings of the treadmill, the angle of the wall sit, and the target rate on the stationary bicycle. The virtual visit protocol was amended to collect additional information about contacts with healthcare providers. We also provided personalized counseling to individuals who developed exertional hypertension during the in-person exercise training sessions to modify the intensity of specific exercises and to minimize Valsalva maneuvers during isometric exercises. New participants in the guided exercise study arm received the updated exercise instructions at the initial enrollment visit. Previously enrolled participants received updated instructions and teaching at virtual follow-up visits.

### Limitations

The principal limitations to study recruitment were the requirements for participants to have access to exercise equipment at home and for travel to in-person study visits. Technological barriers did limit timely virtual follow-up visits with some participants. The follow-up period of this trial is not long enough to determine if exercise can reduce aortic events. We plan to address these obstacles in a larger and longer trial that will be adequately powered to determine if guided exercise can reduce aortic events and prevent deaths due to TAD. In such a trial, we will collect longitudinal data on aortic enlargement, arterial stiffness, cardiac function, and serial changes in blood pressure responses to exercise over time. To promote accessibility, we plan to mail portable exercise equipment directly to participants. In the short term, we plan to adapt this protocol to create personalized exercise prescriptions for patients, and in the long term, we hope that these studies may eventually be used to develop evidence-based exercise guidelines.

### Conclusions

Guided exercise is safe for aortic dissection survivors. Ambulatory blood pressure can predict exertional blood pressure responses and may improve risk stratification and medical optimization of TAD patients who plan to begin an exercise program. Short-term exposure to guided exercise increased confidence and decreased the anxiety of study participants.

## Supplementary Material

Supplementary Material

## Figures and Tables

**Table 1.** Demographic characteristics

Variable	All participants (*n* = 81)	Guided exercise (*n* = 38)	Usual care (*n* = 43)	*P*

**Age (*y*)**	57 (17)	56 (17)	57 (19)	1.0
**Sex**				
Female	22 (27)	11 (29)	11 (26)	0.9
**Ethnicity**				
Hispanic or latino	2 (2)	1 (3)	1 (2)	1.0
**Race**				
American Indian/Alaska Native	2 (3)	0 (0)	2 (5)	0.5
Asian	5 (6)	2 (5)	3 (7)	1.0
Native Hawaiian/other Pacific Islander	1 (1)	0 (0)	1 (2)	1.0
Black or African American	9 (11)	4 (11)	5 (12)	1.0
White	61 (75)	32 (84)	29 (67)	0.1
**Antihypertensive medications**				
Beta blocker ACEi/ARB	75 (93)	37 (97)	38 (88)	0.2
Diuretic	45 (55)	25 (66)	20 (47)	0.1
Calcium channel blocker	22 (27)	13 (34)	9 (21)	0.6
	31 (38)	12 (32)	19 (44)	0.3
**Dissection data**				
Time since dissection (*y*)	3.5 (3)	4 (3)	3.1 (2)	0.7
Type A	51 (63)	25 (66)	26 (60)	0.4
Type B	25 (31)	10 (26)	15 (35)	0.5
Multiple dissections	5 (6)	3 (8)	2 (5)	0.7

Values are mean (interquartile range), *n* (%). ACEi: Angiotensin-converting enzyme inhibitor; ARB: angiotensin II receptor blockers.

**Table 2.** ABPM characteristics by exertional hypertension

Variable	Total (*n* = 70)	Exertional hypertension (*n* = 13)	No exertional hypertension (*n* = 24)	*P*

Mean SBP	119 (16)	122 (16)	114 (13)	0.13
Mean DBP	67 (12)	70 (11)	64 (6)	0.06
DaySBP	123 (18)	127 (14)	119 (16)	0.3
Day DBP	70 (12)	74 (9)	67 (5)	0.05*
Night SBP	111 (16)	111 (19)	103 (17)	0.12
Night DBP	61 (14)	62 (11)	58 (14)	0.22
Peak daytime SBP	157 (28)	178 (34)	154 (22)	0.01*
Pulse pressure	50 (12)	46 (10)	48 (17)	0.76
Daytime SBP COV	11 (4)	14 (4)	11 (4)	0.03*
Morning surge index (%)	16 (18)	18 (28)	18 (17)	0.78
Nocturnal dipping (%)	12 (12)	17 (10)	14 (9)	0.10
AASI	0.53 (0.16)	0.48 (0.08)	0.52 (0.16)	0.51

Values are mean (interquartile range). SBP: Systolic blood pressure; DBP: diastolic blood pressure; PP: pulse pressure; AASI: ambulatory arterial stiffness index; COV: coefficient of variation. Exertional hypertension: SBP > 180 mmHg or DBP > 100 mmHg in > 1 exercise

* ANOVA *P* < 0.05.

## Data Availability

Data will be published as Supplementary Material.

## References

[1] Meinlschmidt G, Berdajs D, Moser-Starck R, Perceived need for psychosocial support after aortic dissection: cross-sectional survey. J Particip Med 2020;12:e15447. 33064108 10.2196/15447 PMC7434062

[2] Ghadieh AS, Saab B. Evidence for exercise training in the management of hypertension in adults. Can Fam Physician 2015;61:233–9. 25927108 PMC4369613

[3] Chaddha A, Eagle KA, Braverman AC, Exercise and physical activity for the post-aortic dissection patient: the clinician’s conundrum. Clin Cardiol 2015;38:647–51. 26769698 10.1002/clc.22481 PMC6490791

[4] Chaddha A, Kline-Rogers E, Braverman AC, Survivors of aortic dissection: activity, mental health, and sexual function. Clin Cardiol 2015;38:652–9. 26769699 10.1002/clc.22418 PMC6490749

[5] Li J, Boyd A, Huang M, Berookhim J, Prakash SK. Safety of exercise for adults with thoracic aortic aneurysms and dissections. Front Sports Act Living 2022;4:888534. 36072558 10.3389/fspor.2022.888534 PMC9441662

[6] Gibson C, Nielsen C, Alex R, Mild aerobic exercise blocks elastin fiber fragmentation and aortic dilatation in a mouse model of Marfan syndrome associated aortic aneurysm. J Appl Physiol 2017;123:147–60. 28385916 10.1152/japplphysiol.00132.2017

[7] Milewicz DM, Prakash SK, Ramirez F. Therapeutics targeting drivers of thoracic aortic aneurysms and acute aortic dissections: insights from predisposing genes and mouse models. Annu Rev Med 2017;68:51–67. 28099082 10.1146/annurev-med-100415-022956 PMC5499376

[8] MacDougall JD, Tuxen D, Sale DG, Moroz JR, Sutton JR. Arterial blood pressure response to heavy resistance exercise. J Appl Physiol 1985;58:785–90. 3980383 10.1152/jappl.1985.58.3.785

[9] Robicsek F, Thubrikar MJ. Hemodynamic considerations regarding the mechanism and prevention of aortic dissection. Ann Thorac Surg 1994;58:1247–53. 7944800 10.1016/0003-4975(94)90523-1

[10] Hamer M, Bauman A, Bell JA, Stamatakis E. Examining associations between physical activity and cardiovascular mortality using negative control outcomes. Int J Epidemiol 2019;48:1161–6. 30541040 10.1093/ije/dyy272 PMC6693890

[11] Chaddha A, Kline-Rogers E, Woznicki EM, Cardiology patient page. Activity recommendations for postaortic dissection patients. Circulation 2014;130:e140–2. 25311622 10.1161/CIRCULATIONAHA.113.005819

[12] Nakayama A, Morita H, Nagayama M, Cardiac rehabilitation protects against the expansion of abdominal aortic aneurysm. J Am Heart Assoc 2018;7:e007959. 29487112 10.1161/JAHA.117.007959 PMC5866332

[13] Corone S, Iliou MC, Pierre B, French registry of cases of type I acute aortic dissection admitted to a cardiac rehabilitation center after surgery. Eur J Cardiovasc Prev Rehabil 2009;16:91–5. 19237998 10.1097/HJR.0b013e32831fd6c8

[14] Hornsby WE, Norton EL, Fink S, Cardiopulmonary exercise testing following open repair for a proximal thoracic aortic aneurysm or dissection. J Cardiopulm Rehabil Prev 2020;40:108–15. 31478921 10.1097/HCR.0000000000000446 PMC7048630

[15] Ritchey MD, Maresh S, McNeely J, Tracking cardiac rehabilitation participation and completion among medicare beneficiaries to inform the efforts of a national initiative. Circ Cardiovasc Qual Outcomes 2020;13:e005902. 31931615 10.1161/CIRCOUTCOMES.119.005902 PMC8091573

[16] Muntner P, Shimbo D, Carey RM, Measurement of blood pressure in humans: a scientific statement from the american heart association. Hypertension 2019;73:e35–66. 30827125 10.1161/HYP.0000000000000087 PMC11409525

[17] Lewis JE, Nash MS, Hamm LF, Martins SC, Groah SL. The relationship between perceived exertion and physiologic indicators of stress during graded arm exercise in persons with spinal cord injuries. Arch Phys Med Rehabil 2007;88:1205–11. 17826469 10.1016/j.apmr.2007.05.016

[18] Dolan E, Thijs L, Li Y, Ambulatory arterial stiffness index as a predictor of cardiovascular mortality in the Dublin Outcome Study. Hypertension 2006;47:365–70. 16432047 10.1161/01.HYP.0000200699.74641.c5

[19] Kario K Evidence for the surge blood pressure resonance hypothesis as a trigger for cardiovascular disease events. Hypertens Res 2023;46:2065–9. 37322131 10.1038/s41440-023-01346-3

[20] Liu W, Leong DP, Hu B, The association of grip strength with cardiovascular diseases and all-cause mortality in people with hypertension: findings from the prospective urban rural epidemiology china study. J Sport Health Sci 2021;10:629–36. 33091627 10.1016/j.jshs.2020.10.005 PMC8724607

[21] Leong DP, Teo KK, Rangarajan S, Prognostic value of grip strength: findings from the prospective urban rural epidemiology (PURE) study. Lancet 2015;386:266–73. 25982160 10.1016/S0140-6736(14)62000-6

