# Supplementary material for: Multicenter randomized controlled trial of exercise in aortic dissection survivors: rationale, design, and initial hemodynamic data
Source: Vessel Plus. Author manuscript; Available in PMC 2026 Mar 3. (PMC12952786; doi:10.20517/2574-1209.2023.149)
Supplement: Supplementary Material [file NIHMS2133590-supplement-Supplementary_Material.docx]

**Supplementary Material: Multicenter Randomized Controlled Trial of Exercise in Aortic Dissection Survivors: Rationale, Design, and Initial Hemodynamic Data**

Supplementary Table 1. Most frequently observed Space Labs ABPM error codes.

| Error Code | Condition |
| --- | --- |
| EC04 | Occasional EC04 messages reflect excessive patient movement. Frequent EC04 messages indicate an improperly applied cuff or a monitor malfunction |
| EC10, 70, 90 | Excessive movement |
| EC11 | Monitor did not pump above mean arterial level |
| EC40 | Movement during systole |
| EC50, 58 | Movement during diastole |
| EC52 | Kinked tubing |
| EC62 | Cuff applied too loosely |

**Supplemental Text 1. BRFSS Questionnaire**

**BRFSS Questionnaire: Physical Activity**

When you are at work, which of the following best Mostly sitting or standing describes what you do? Mostly walking

Mostly heavy labor or physically demanding work


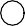

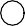

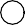

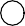


If you have more than one job, consider all jobs in Don't know / Not sure your answer. (include all jobs)


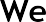

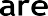

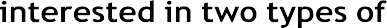

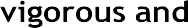

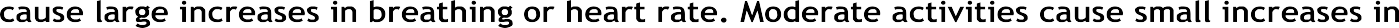

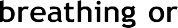


Thinking about the moderate activities you do in a Yes

usual week, do you do moderate activities for at least No


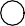

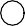

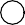


10 minutes at a time, such as brisk walking, Don't know / Not sure bicycling, vacuuming, gardening, or anything else that

causes a small increase in breathing or heart rate?

How many days per week do you do these moderate

activities for at least 10 minutes at a time?

On days when you do moderate activities for at least

10 minutes at a time, how much total time per day do you spend doing these activities? Answer to the

nearest hour.

Now, thinking about the vigorous activities you do in Yes

a usual week, do you do vigorous activities for at No


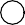

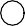

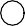


least 10 minutes at a time, such as running, aerobics, Don't know / Not sure heavy yard work, or anything else that causes large

increases in breathing or heart rate?

How many days per week do you do these vigorous

activities for at least 10 minutes at a time?

On days when you do vigorous activities for at least

10 minutes at a time, how much total time per day do you spend doing these activities? Answer to the

nearest hour.

For BRFSS Survey Questions

Centers for Disease Control and Prevention (CDC). Behavioral Risk Factor Surveillance System Survey Questionnaire.

Atlanta, Georgia: U.S. Department of Health and Human Services, Centers for Disease Control and Prevention, 2009.<http://www.cdc.gov/brfss/suggestedcitation.htm>

Supplemental Text 2. PROMIS Questionnaires

1. **PROMIS - Physical Function**

Please complete the survey below. Thank you!

T-score

Standard Error

Are you able to do chores such as vacuuming or yard Without any difficulty work? With a little difficulty

With some difficulty With much difficulty Unable to do


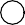

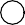

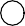

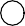

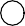


Are you able to go up and down stairs at a normal Without any difficulty

pace? With a little difficulty


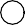

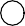

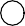

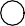

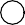


With some difficulty With much difficulty Unable to do

Are you able to go for a walk of at least 15 minutes? Without any difficulty

With a little difficulty With some difficulty With much difficulty Unable to do


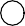

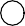

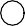

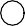

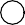

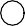

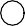

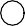

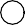

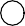


Are you able to run errands and shop? Without any difficulty With a little difficulty With some difficulty With much difficulty Unable to do

Acknowledgment: PROMIS Health Organization and Assessment Center℠ View full acknowledgment

### PROMIS - Anxiety

T-score

Standard Error

In the past 7 days Never

I felt fearful Rarely


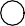

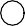

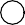

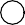

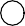


Sometimes Often Always

In the past 7 days Never

I found it hard to focus on anything other than my Rarely


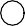

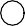

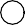

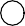

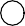


anxiety Sometimes

Often Always

In the past 7 days Never

My worries overwhelmed me Rarely


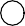

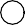

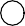

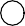

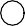


Sometimes Often Always

In the past 7 days Never

I felt uneasy Rarely


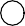

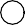

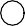

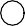

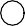


Sometimes Often Always

Acknowledgment: PROMIS Health Organization and Assessment Center℠ View full acknowledgment

### PROMIS - Depression

T-score

Standard Error

In the past 7 days Never

I felt worthless Rarely


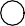

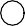

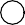

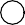

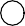


Sometimes Often Always

In the past 7 days Never

I felt helpless Rarely


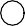

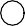

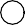

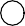

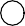


Sometimes Often Always

In the past 7 days Never

I felt depressed Rarely


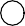

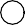

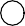

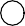

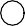


Sometimes Often Always

In the past 7 days Never

I felt hopeless Rarely


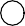

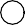

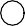

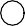

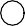


Sometimes Often Always

Acknowledgment: PROMIS Health Organization and Assessment Center℠ View full acknowledgment

### PROMIS - Fatigue

T-score

Standard Error


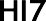


During the past 7 days: Not at all

I feel fatigued A little bit


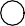

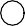

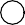

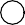

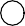


Somewhat Quite a bit Very much

In the past 7 days Not at all

How fatigued were you on average? A little bit Somewhat Quite a bit Very much


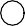

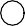

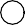

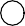

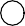


In the past 7 days Not at all

How run-down did you feel on average? A little bit Somewhat Quite a bit Very much


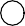

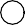

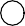

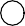

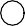


During the past 7 days: Not at all

I have trouble starting things because I am tired A little bit Somewhat Quite a bit Very much


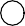

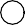

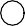

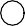

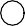


Acknowledgment: PROMIS Health Organization and Assessment Center℠ View full acknowledgment

### PROMIS - Sleep Disturbance

T-score

Standard Error

In the past 7 days Not at all

My sleep was refreshing. A little bit


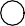

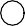

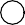


Somewhat Quite a bit Very much

In the past 7 days Not at all

I had a problem with my sleep. A little bit Somewhat Quite a bit Very much

In the past 7 days Not at all

I had difficulty falling asleep. A little bit

Somewhat Quite a bit Very much

In the past 7 days Very poor

My sleep quality was... Poor

Fair Good

Very good

Acknowledgment: PROMIS Health Organization and Assessment Center℠ View full acknowledgment

### PROMIS - Ability to Participate Social

T-score

Standard Error

I have trouble doing all of my regular leisure Never

activities with others Rarely

Sometimes Usually Always

I have trouble doing all of the family activities that Never

I want to do Rarely

Sometimes Usually Always

I have trouble doing all of my usual work (include Never

work at home) Rarely

Sometimes Usually Always

I have trouble doing all of the activities with Never

friends that I want to do Rarely

Sometimes Usually Always

Acknowledgment: PROMIS Health Organization and Assessment Center℠ View full acknowledgment

### PROMIS - Pain Interference

T-score

Standard Error

In the past 7 days Not at all

How much did pain interfere with your day to day A little bit

activities? Somewhat

Quite a bit Very much

In the past 7 days Not at all

how much did pain interfere with work around the home? A little bit

Somewhat Quite a bit Very much

In the past 7 days Not at all

How much did pain interfere with your ability to A little bit

participate in social activities? Somewhat Quite a bit Very much

In the past 7 days Not at all

How much did pain interfere with your household A little bit

chores? Somewhat

Quite a bit Very much

Acknowledgment: PROMIS Health Organization and Assessment Center℠ View full acknowledgment

Supplemental Text 3. Clinical Trial Evaluation Survey

**Evaluation Survey**

Please complete the survey below. Thank you!

1. How did you generally feel about engaging in moderate physical activity?

Confident Neutral Uneasy

*(Place a mark on the scale above)*

1. How would you rate your confidence to engage in physical activity?

Moderate

High confidence confidence Low confidence

*(Place a mark on the scale above)*

1. How would you rate your outlook on exercise post-dissection?

Excited Neutral Discouraged

*(Place a mark on the scale above)*

1. How do you generally feel about engaging in moderate physical activity?

Confident Neutral Uneasy

*(Place a mark on the scale above)*

1. How would you rate your confidence to engage in physical activity?

Moderate

High confidence confidence Low confidence

*(Place a mark on the scale above)*

1. How would you rate your outlook on exercise post-dissection?

Excited Neutral Discouraged

*(Place a mark on the scale above)*

1. Participating in the clinical trial has positively Strongly Disagree changed my outlook on exercise post-dissection. Disagree

Neither agree nor disagree Agree

Strongly Agree

1. Participating in the clinical trial has not changed my Strongly Disagree outlook on exercise-on-exercise post-dissection. Disagree

Neither agree nor disagree Agree

Strongly Agree

1. Participating in the clinical trial has negatively Strongly Disagree changed my outlook on exercise post-dissection. Disagree

Neither agree nor disagree Agree

Strongly Agree

1. Please use this field to provide any feedback about your experiences with the clinical trial thus far.

Supplementary Table 2. Demographic Characteristics by Intervention Type

| Variable | Participants (*n* = 81) | TEVAR (*n* = 12) | Open Repair (*n* = 53) | | No Intervention (*n* = 16) | |
| --- | --- | --- | --- | --- | --- | --- |
| Age (y) | 56.6 (16.8) | 52.5 (14.1) | | 57.6 (11) | | 56.5 (22.1) |
| Sex  Female | 22 (27) | 2 (17) | | 14 (26) | | 6 (38) |
| Antihypertensive Medications  Beta blocker  ACEi/ARB  Diuretic  Calcium channel blocker | 2 (1)  75 (93)  45 (56)  22 (27)  31 (38) | 3 (3)  12 (100)  8 (67)  5 (42)  7 (58) | | 2 (1)  49 (92)  30 (57)  14 (26)  17 (32) | | 2 (1)  14 (88)  7 (44)  3 (19)  7 (44) |
| Dissection Data  Time since dissection (y)  Type A  Type B  Multiple dissections | 3.5 (3)  51 (63)  25 (31)  5 (6) | 2.2 (3)  2 (17)  10 (83)  0 (0) | | 3.8 (3)  44 (83)  4 (8)  5 (9) | | 3.9 (3)  5 (31)  11 (69)  0 (0) |

Values are mean (interquartile range), n (%). ACEi: angiotensin-converting enzyme inhibitor. ARB: angiotensin II receptor blockers.

**Supplementary Table 3. ABPM Characteristics by Intervention Type**

| **Variable** | **Total**  **(n=70)** | **TEVAR (n = 10)** | **Open Repair (n=47)** | **No Intervention (n=13)** | ***P*** |
| --- | --- | --- | --- | --- | --- |
| **Mean SBP** | 119 (16) | 121 (7) | 118 (16) | 121 (20) | 0.9 |
| **Mean DBP** | 67 (12) | 69 (10) | 66 (11) | 69 (14) | 0.7 |
| **Day SBP** | 123 (18) | 124 (11) | 122 (18) | 124 (14) | 0.9 |
| **Day DBP** | 70 (12) | 71 (12) | 68 (11) | 71 (11) | 0.7 |
| **Night SBP** | 111 (16) | 113 (17) | 109 (16) | 113 (15) | 0.8 |
| **Night DBP** | 61 (14) | 64 (12) | 60 (11) | 63 (14) | 0.5 |
| **Peak daytime SBP** | 157 (28) | 149 (7) | 158 (27) | 153 (28) | 0.6 |
| **Pulse Pressure** | 50 (12) | 52 (11) | 52 (12) | 52 (11) | 0.9 |
| **Daytime SBP COV** | 11 (4) | 10 (1.5) | 11 (5) | 9 (2) | 0.08 |
| **Morning Surge Index (%)** | 16 (18) | 21 (7) | 17 (16) | 12 (23) | 0.4 |
| **Nocturnal Dipping (%)** | 12 (12) | 7 (7) | 13 (9) | 11 (13) | 0.3 |
| **AASI** | 0.53 (0.16) | 0.6 (0.20) | 0.53 (0.17) | 0.48 (0.15) | 0.2 |

Values are mean (interquartile range). SBP: systolic blood pressure; DBP: diastolic blood pressure; PP: pulse pressure; AASI: ambulatory arterial stiffness index; COV: coefficient of variation; *: ANOVA *P* < 0.05.

**PROMIS Scores by Intervention Type**

Evaluation of seven PROMIS domains in patients who received no intervention found that mean T scores for anxiety (52 ± 10), pain (51 ± 8), and impairment of participation in social activities (54 ± 8) were increased. Similarly, for patients who received open repair, the mean T scores for anxiety (52 ± 8), pain (51 ± 7), and impairment of participation in social activities (54 ± 8) were also increased. For participants who received TEVAR, the mean T scores for pain (52 ± 8) and impairment of participation in social activities (55 ± 7) were increased. All other PROMIS domains were within normal limits for each group.

**Grip Strength by Intervention Type**

At baseline, the mean maximum grip strength for the no intervention, open repair, and TEVAR groups were as follows: 58.6 lbs (IQR 4.3), 66.4 lbs (IQR 14.35), 62.5 lbs (IQR 3.2).

**Orthostasis by Intervention Type**

At baseline, 20% of patients who received no intervention, 4% of patients who received open repair, and 23% of patients who received TEVAR exhibited postural orthostasis.

**Exertional Hypertension by Intervention Type**

15 participants exhibited exertional hypertension. One patient in the open repair group developed severe exertional hypertension >210 mmHg. Exercises that caused SBP to exceed 180 mmHg for the no intervention group were: wall sits (50%), stationary bicycling (50%), and treadmill (50%). For the open repair group, exercises that caused SBP to exceed 180 mmHg were: bicep curls (27%), wall sits (55%), leg raise (1%), stationary bicycling (18%), and treadmill (1%). For the TEVAR group, exercises that caused SBP to exceed 180 mmHg were: hand grip (50%), stationary bicycling (50%), and treadmill (50%).
